# Prediction of sarcopenia using a combination of multiple serum biomarkers

**DOI:** 10.1038/s41598-018-26617-9

**Published:** 2018-06-05

**Authors:** Ju Yeon Kwak, Hyeoncheol Hwang, Seon-Kyu Kim, Jeong Yi Choi, Seung-Min Lee, Hyun Bang, Eun-Soo Kwon, Kwang-Pyo Lee, Sun Gun Chung, Ki-Sun Kwon

**Affiliations:** 1 Aging Research Center, Korea Research Institute of Bioscience & Biotechnology (KRIBB), 125 Gwahak-ro, Yuseong-gu, Daejeon, 34141 Republic of Korea; 2 Department of Rehabilitation Medicine, Seoul National University College of Medicine, Seoul National University Hospital, 101 Daehak-ro, Jongno-gu, Seoul, 03080 Republic of Korea; 3 Personalized Genomic Medicine Research Center, Korea Research Institute of Bioscience & Biotechnology (KRIBB), 125 Gwahak-ro, Yuseong-gu, Daejeon, 34141 Republic of Korea; 4 Institute of Aging, Seoul National University, 101 Daehak-ro, Jongno-gu, Seoul, 03080 Republic of Korea; 5 Department of Biomolecular Science, KRIBB School of Bioscience, Korea University of Science and Technology (UST), 217 Gajeong-ro, Yuseong-gu, Daejeon, 34113 Republic of Korea; 6 Department of Functional Genomics, KRIBB School of Bioscience, Korea University of Science and Technology (UST), 217 Gajeong-ro, Yuseong-gu, Daejeon, 34113 Republic of Korea

## Abstract

Sarcopenia is a gradual loss of skeletal muscle mass and function with aging. Given that sarcopenia has been recognized as a disease entity, effective molecular biomarkers for early diagnosis are required. We recruited 46 normal subjects and 50 patients with moderate sarcopenia aged 60 years and older. Sarcopenia was clinically identified on the basis of the appendicular skeletal muscle index by applying cutoff values derived from the Asian Working Group for Sarcopenia. The serum levels of 21 potential biomarkers were analyzed and statistically examined. Interleukin 6, secreted protein acidic and rich in cysteine, macrophage migration inhibitory factor, and insulin-like growth factor 1 levels differed significantly between the normal and sarcopenia groups. However, in each case, the area under the receiver operating characteristics curve (AUC) was <0.7. Subsequent combination of the measurements of these biomarkers into a single risk score based on logistic regression coefficients enhanced the accuracy of diagnosis, yielding an AUC value of 0.763. The best cutoff value of 1.529 had 70.0% sensitivity and 78.3% specificity (95% CI = 2.80–21.69, p < 0.0001). Combined use of the selected biomarkers provides higher diagnostic accuracy than individual biomarkers, and may be effectively utilized for early diagnosis and prognosis of sarcopenia.

## Introduction

Sarcopenia, defined as age-related decline in skeletal muscle mass and function^1^, is the most significant cause of frailty in the elderly^2^. The recent upsurge of research on sarcopenia has supported the establishment of a new disease code in ICD-10-CM^3^. Therefore, effective diagnostic tools that could facilitate early diagnosis and prognosis of sarcopenia remain an urgent unmet medical need. Diagnostic criteria for sarcopenia are based on low muscle mass and function (strength or performance)^4^. A number of technologies are currently employed to estimate muscle mass, including magnetic resonance imaging (MRI), computed tomography (CT), dual energy X-ray absorptiometry scans (DXA) and bioelectrical impedance analysis (BIA). Imaging-related quantification using MRI and CT has been considered as gold standard to measure muscle mass, but have drawbacks of high cost, low accessibility and CT-generated radiation exposure^5^. DXA is widely used due to minimal radiation exposure and lower cost than MRI and CT^6^. BIA is simple and portable, in spite of less reliability than imaging modalities owing to the influence of various factors, such as ethnicity, gender, age, and hydration status^7^. Physical function tests, such as grip strength and gait speed, may be disrupted by comorbidities, such as joint problems and neurologic deficits, which prevail in population with sarcopenia. In addition, current methods to diagnose sarcopenia are operational only after the onset or progression of disease (e.g., loss of muscle mass or mobility disability). Therefore, determination of efficient molecular biomarkers for early diagnosis and prognosis of sarcopenia in routine clinical practice is essential^8^.

Several mechanisms are potentially involved in pathogenesis of sarcopenia. Both intrinsic (e.g., inflammation, apoptosis, autophagy, mitochondria, neuromuscular junction, calcium metabolism) and extrinsic (e.g., endocrine, nutritional status, immobility) factors^9–14^ contribute to defective myogenesis, muscle atrophy and weakness. Considering the multi-layered mechanisms underlying sarcopenia, no single biomarker may accurately represent this state. Here, we performed a comparative analysis of potential biomarkers between sarcopenia and normal elderly groups with the aim of identifying multiple serum biomarkers. We further combined the measurements of multiple biomarker candidates via logistic regression to improve diagnostic accuracy for sarcopenia.

## Materials and Methods

### Subjects and samples

Eligible candidates were community-dwelling adults aged 60 years and older. Candidates were recruited from several senior centers or halls for the elderly in Seoul. Individuals with a history of alcohol abuse or drug overuse, insulin-dependent diabetes mellitus, phenylketonuria, current heavy smokers (>1 pack/day) and amputees were excluded from study. Appendicular skeletal muscle mass (ASM) was measured using bioelectrical impedance analysis (BIA). Grip strength of the dominant hand and 4 m walking speed^15^ were additionally evaluated.

Subjects were clinically classified as moderate sarcopenia according to appendicular skeletal muscle index (ASMI; ASM/height2) <7.0 kg/m^2^ for men and <5.7 kg/m^2^ for women, as recommended by the Asian Working Group for Sarcopenia (AWGS)^16^. In total, 50 sarcopenic and 46 normal subjects participated in the study. Blood samples were collected in serum separator tubes, frozen and stored until biomarker analysis.

The Institutional Review Board of Seoul National University Hospital approved all procedures, and all methods were performed in accordance with the relevant guidelines and regulations. All participants provided written informed consent upon enrollment.

### Enzyme-linked immunosorbent assay (ELISA)

Serum levels of human IL-6, SPARC, MIF, and IGF-1 were measured using ELISA kits according to the manufacturer’s protocol (R&D systems, Inc., Minneapolis, MN, USA).

### Development of risk score

To develop a single risk score using all four biomarkers (IL-6, SPARC, MIF and IGF-1), we adopted a previously developed strategy with regression analysis for multiple biomarkers^17,18^. Briefly, serum levels were log_2_ transformed to reduce variations between the concentrations of each biomarker and used to generate logistic regression coefficients. The risk score for each individual was calculated as the sum of the risk score for each biomarker, which was derived by multiplying the serum level of a biomarker by its corresponding coefficient (Risk score = ∑ logistic regression coefficient of biomarker *M*_*i*_ × serum level of biomarker *M*_*i*_). Subjects were subsequently divided into two groups (high and low risk of sarcopenia) using the median cutoff risk score as a threshold.

### Statistics

The significance of association between a risk group and clinically manifested sarcopenia was assessed using Fisher’s exact test. To estimate the utility of risk score as a diagnostic tool of sarcopenia, we calculated the area under curve (AUC) value using receiver operating characteristics (ROC) analysis. The significance of serum level differences between normal and sarcopenia groups was assessed using two-sample t-tests for each biomarker. Data were considered statistically significant at p-values < 0.05 and false discovery rate <0.3. All statistical analyses were performed using GraphPad Prism5 (GraphPad Software, Inc., USA) and R language environment (ver. 3.2.5).

## Results

### Baseline characteristics

Mean appendicular skeletal muscle index (ASMI) values of normal (46 subjects) and sarcopenia (50 subjects) groups were 7.50 kg/m^2^ and 6.51 kg/m^2^ respectively in men, and 6.48 kg/m^2^ and 5.23 kg/m^2^ respectively in women. The sarcopenia group had weaker grip strength than the normal group (27.83 kgF vs 32.28 kgF in men and 17.06 kgF vs 20.86 kgF in women) but displayed no significant differences in gait speed. The baseline characteristics are shown in Table 1.

**Table 1:** Baseline characteristics of the samples.

	Men		*P*-value^*^	Women		*P*-value^*^
	Normal	Sarcopenia		Normal	Sarcopenia	
Number of subjects	18	18		28	32	
Age (years)^**^	76.00 ± 5.72	76.22 ± 5.62	0.907	71.86 ± 6.01	76.13 ± 6.05	0.008
Height (cm)^**^	164.16 ± 5.02	161.56 ± 3.97	0.093	152.90 ± 5.27	147.14 ± 5.14	<0.001
Weight (kg)^**^	65.68 ± 6.05	57.47 ± 6.29	<0.001	62.40 ± 6.58	49.85 ± 5.95	<0.001
Appendicular skeletal muscle mass (kg)^**^	20.23 ± 2.09	16.97 ± 1.86	<0.001	15.23 ± 1.67	11.38 ± 1.29	<0.001
Appendicular skeletal muscle index (km/m^2^)^**^	7.50 ± 0.41	6.51 ± 0.54	<0.001	6.48 ± 0.39	5.23 ± 0.30	<0.001
Grip strength (kgF)^**^	32.28 ± 6.50	27.83 ± 5.09	0.029	20.86 ± 4.91	17.06 ± 2.71	<0.001
Gait speed (m/s)^**^	0.99 ± 0.14	1.01 ± 0.16	0.685	1.00 ± 0.22	0.98 ± 0.15	0.741

*Independent t-test.

**Mean ± standard deviation.

### Selection of biomarkers for diagnosis of sarcopenia

To identify effective molecular biomarkers for diagnosis of sarcopenia, candidates were selected by searching the relevant literature and subjected to immunochemical analyses using elderly people sera of normal and sarcopenia group that was clinically manifested based on AWGS. We examined 21 biomarker candidates, including proteins related to skeletal muscle function or metabolism and muscle-derived cytokines (myokines), angiotensin-converting enzyme (ACE)^19^, insulin-like growth factor 1 (IGF-1)^20^, procollagen type III N-terminal peptide (P3NP)^21^, fibroblast growth factor 21 (FGF21)^22^, myostatin^23^, growth differentiation factor 11 (GDF11)^24^, meteorin-like (METRNL)^25^, macrophage migration inhibitory factor (MIF)^26^, fatty acid binding protein 3 (FABP3)^27^, ciliary neurotrophic factor (CNTF)^28^, secreted protein acidic and rich in cysteine (SPARC)^29^, brain-derived neurotrophic factor (BDNF), tumor necrosis factor-α (TNF-α), interleukin 6 (IL-6), interleukin 8 (IL-8), interleukin 10 (IL-10), interleukin 15 (IL-15), interleukin 1β (IL-1β), monocyte chemotactic Protein 1 (MCP-1), transforming growth factor β 1 (TGFβ1), and vascular endothelial growth factor (VEGF)^30^.

**Figure 1:**
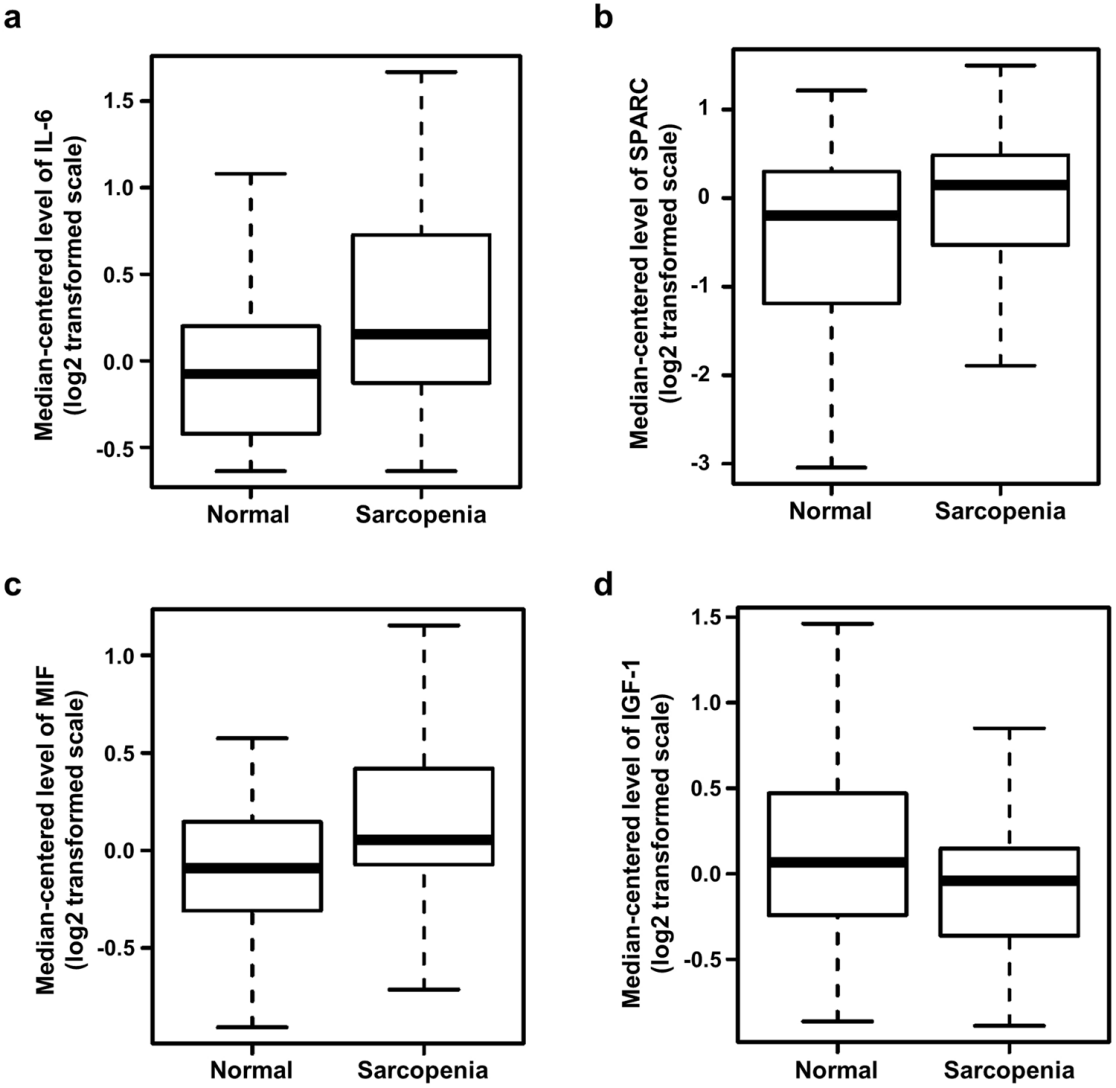
Comparison of serum protein levels between normal and sarcopenic aged subjects. IL-6 (**a**), SPARC (**b**), MIF (**c**) and IGF-1 (**d**) protein levels in human serum were measured using sandwich ELISA. Box plots were used to visualize distribution of each serum protein level. *P*-values were obtained with two sample t-tests.

Among the candidates investigated, serum levels of IL-6, SPARC, MIF and IGF-1 were significantly different between normal and sarcopenia groups (Fig. 1), but not the other biomarkers (data not shown). Serum levels of IL-6 were higher in the sarcopenia than the normal group (Fig. 1a; 1.64 ± 0.36 versus 0.86 ± 0.25 pg/ml, p = 0.015). Exceptionally, the IL-6 levels in three sarcopenia and six normal samples were undetectable with a commercially available ELISA kit that can detect concentrations as low as 0.1 pg/mL. Similarly, serum concentrations of SPARC and MIF were higher in the sarcopenia than the normal group (Fig. 1b,c; 531.5 ± 40.65 versus 409.4 ± 35.47 pg/mL SPARC, p = 0.047, and 25.10 ± 1.19 versus 20.71 ± 0.89 ng/mL MIF, p = 0.008). Conversely, serum IGF-1 levels were lower in the sarcopenia group (Fig. 1d; 58.16 ± 3.37 versus 72.61 ± 5.49, p = 0.039). Based on the collective results, IL-6, SPARC, MIF and IGF-1 were selected for analysis as potential biomarkers for sarcopenia.

### Application of risk score to predict sarcopenia

**Figure 2:**
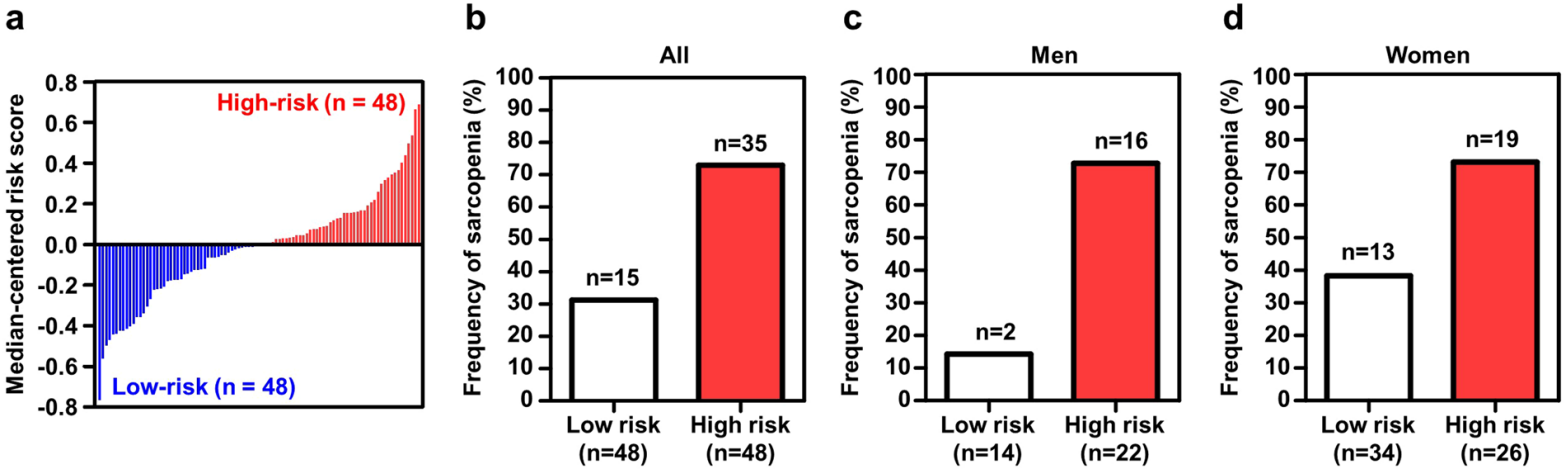
Risk score based on combination of four biomarkers (IL6, SPARC, MIF, and IGF-1) and frequency of sarcopenia. Bar graph of risk scores in 96 samples (**a**). Median risk score across samples was applied as a threshold for dividing into two risk groups (cutoff value = 1.518). Comparison of frequency of sarcopenia between low-risk and high-risk groups in whole population (**b**), Men (**c**), Women (**d**). *P*-values and confidence intervals (CI) were calculated with Fisher’s exact test.

Combination of measurements of multiple biomarkers into a single score was required to integrate the collected information for easy diagnostic judgement. A risk score was generated by combination of the measurements of the four selected biomarkers (IL-6, SPARC, MIF and IGF-1) based on the logistic regression coefficient of each biomarker (Table 2). Using the median cutoff value of risk scores for all subjects (1.518), two groups with high-risk and low-risk scores were classified (Fig. 2a). The frequency of clinically identified sarcopenia was significantly higher in the high-risk than the low-risk group (Fig. 2b; p = 0.002, 95% confidence interval (CI) = 1.56–11.59), indicating that biomarker analysis is clinically valuable for sarcopenia diagnosis. When adapted to groups of men and women separately, biomarker analysis was successfully applied to identify sarcopenia in both genders, representing no gender differences in positive predictive value (p = 0.001, 95% CI = 2.28–171.69 in men; p = 0.009, 95% CI = 1.28–15.66 in women; Fig. 2c,d). However, the frequency of sarcopenia in the low-risk group was lower in men than women, signifying that the negative predictive value is higher in men.

**Table 2:** Univariate logistic regression analyses of 4 proteins.

Protein	Estimated β	Std. Error	P-value
IL-6	0.151	0.062	0.016
SPARC	0.102	0.05	0.047
MIF	0.279	0.102	0.008
IGF-1	−0.129	0.062	0.041

^*^β indicates regression coefficient value.

### Significance of multiple biomarkers for diagnosis of sarcopenia

**Figure 3:**
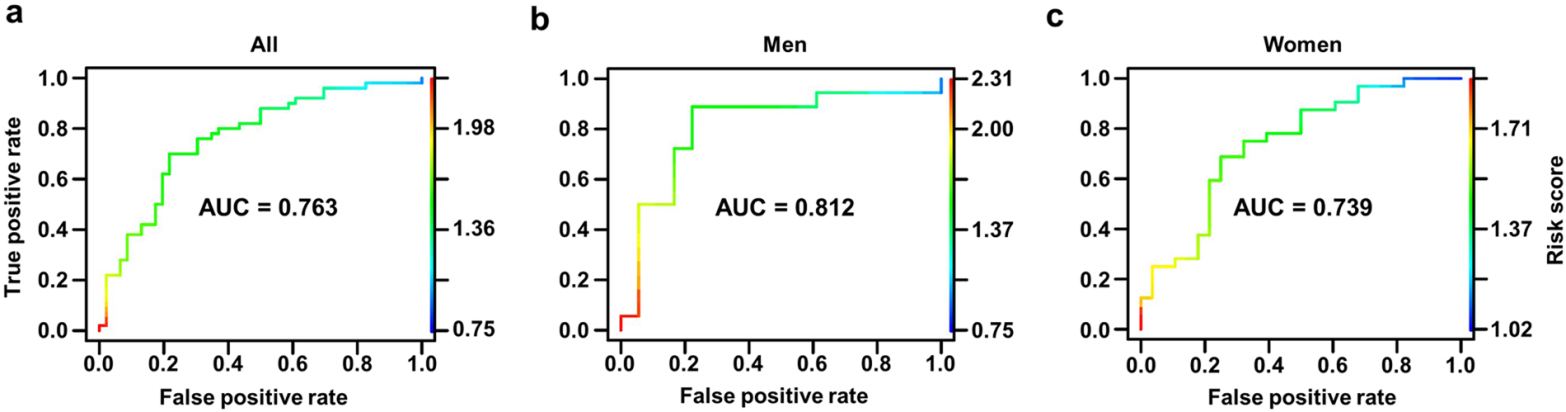
Significance of risk score based on multiple biomarkers for diagnosis of sarcopenia. Receiver operating characteristic (ROC) curves for each group. Whole population (**a**), Men (**b**), Women (**c**). The area under the ROC curve (AUC) was calculated for each group to determine the significance of multiple biomarkers in predicting sarcopenia.

To establish whether the risk score based on multiple biomarkers is useful for diagnosis of sarcopenia, area under the ROC curve, sensitivity and specificity were assessed. When applying ROC analysis to risk score, significantly high AUC values were obtained for all population categories (AUC > 0.7). AUC was 0.763 in the overall elderly population and the best cutoff value, defined as the point with highest multiplicity of sensitivity and specificity, was a risk score of 1.529 (Fig. 3a; 70% sensitivity and 78.26% specificity). AUC was 0.812 and the best cutoff value in elderly men (Fig. 3b; 88.88% sensitivity, 77.77% specificity) was a risk score of 1.543. In elderly women, AUC was 0.739 and a risk score of 1.505 (Fig. 3c; 68.75% sensitivity, 75% specificity) was the best cutoff value. To ascertain whether the optimal cutoff value based on ROC analysis improves predictability, subjects were dichotomized into two groups based on the above cutoff values of risk score. When applying the cutoff values of 1.529, 1.543, and 1.505 to the whole population, men and women groups, respectively, performance in classifying the high-risk sarcopenia group was improved (Supplementary Fig. 1). Our results collectively suggest that the risk score based on multiple biomarkers selected in this study demonstrates predictive value for sarcopenia regardless of gender.

## Discussion

While several candidate molecular biomarkers of sarcopenia have been proposed to date, none have been assessed for predictive ability. The four biomarkers analyzed in this study were not appropriate as single biomarkers for diagnosis of sarcopenia. Despite differences in serum levels between normal and sarcopenia groups with acceptable p-values (Fig. 1), none attained optimal cutoff values at which both sensitivity and specificity were >70%, resulting in AUC <0.7 (data not shown). Interestingly, however, combination of the biomarker measurements into a single risk score enhanced the accuracy of diagnosis for sarcopenia.

Sarcopenia group in our study involved elderly people with lower muscle mass (on the basis of AWGS), but excluded subjects with severe muscle weakness^5^ (grip strength <26 kgF for men and <18 kgF for women and gait speed <0.8 m/s)^16^ and potential accompanying diseases. This ‘moderate sarcopenia’ group showed slightly weaker muscle strength than the normal group but greater strength than the standard sarcopenia group (Table 1). Therefore, selected multiple biomarkers in combination may be effectively utilized for early diagnosis of sarcopenia before clinical manifestation of symptoms, such as weakness, disability, falls, loss of independence, and frailty.

The above biomarkers are evidently correlated with skeletal muscle metabolism and function. IL-6, a well-known pro-inflammatory cytokine, was the first identified myokine^31^. Earlier studies have reported that serum IL-6 is elevated chronically in cachexia and Duchenne muscular dystrophy (DMD) patients and acutely after exercise, and decreases rapidly within an hour to pre-exercise levels^31^. Serum IL-6 has been highlighted as a potential sarcopenia biomarker by several groups, but is yet to be statistically validated.

SPARC, also known as osteonectin or basement-membrane protein 40, is an acidic extracellular matrix glycoprotein secreted by most tissues and involved in bone mineralization and cell-matrix interactions^32–34^. SPARC is highly expressed in patients with various types of myopathy including DMD, inclusion body myositis, and congenital muscular dystrophy^35^. Controversially, another study showed that SPARC overexpression extracellularly inhibits muscle cell differentiation^36^, and its deficiency in mouse skeletal muscle causes myofiber atrophy^37^. Although reduced levels of SPARC in aged muscle may correlate with sarcopenia^29^, changes in the circulating levels of SPARC with aging have not been documented.

MIF, a pro-inflammatory cytokine, has been implicated in muscle damage, in view of its increased levels in muscle of polymyositis patients^26^. Since MIF is suggested to regulate glucose homeostasis^38^ and skeletal muscle is a major organ for glucose utilization, we speculate that circulating MIF levels may reflect the status of glucose metabolism in sarcopenia.

IGF-1, also known as somatomedin C, is a well-known mediator of muscle growth and subsequent regeneration^39^. IGF-1 signaling involving the activation of phosphatidylinositol-3-kinase and Akt (or protein kinase B) not only induces muscle hypertrophy via stimulation of protein synthesis pathways but also predominantly prevents activation of muscle atrophy pathways via inhibition of forkhead box O (FoxO) transcription factors^40^. IGF-1 is produced by satellite cells in regenerating skeletal muscles^41^ and muscle-specific expression of the protein accelerates regeneration of injured muscle by rapidly modulating the inflammatory response^42^. Furthermore, IGF-1 triggers human myotube hypertrophy by accelerating the recruitment of reserve cells in human skeletal muscle^43^.

Effective multiple biomarkers for sarcopenia diagnosis are not confined to the four biomarkers assessed in this study. To reflect entire biological pathways associated with sarcopenia disease activity, optimal combinations of additional novel biomarkers require exploration. Furthermore, longitudinal studies are essential to determine whether analysis of multiple biomarkers can be employed for not only early diagnosis but also evaluating therapeutic interventions and prognosis prior to changes in muscle mass, strength or function. In addition, considering that inflammation is a common mechanism in a variety of diseases associated with aging, such as cancer and diabetes in addition to sarcopenia, specific biomarkers should exclude the possibility of interference by other diseases^44^.

Despite the limitations of the current study, our results clearly support a new innovative approach in molecular diagnosis and prognosis of sarcopenia using multiple biomarkers and provide a step in further understanding the mechanisms underlying sarcopenia and identifying novel therapeutic targets for improving health and quality of life in the elderly.

## Electronic supplementary material

Supplementary Figure 1.

## References

[1] Rosenberg IH (1997). Sarcopenia: origins and clinical relevance. J Nutr.

[2] Roubenoff R (2000). Sarcopenia: a major modifiable cause of frailty in the elderly. J Nutr Health Aging.

[3] Cao L, Morley JE (2016). Sarcopenia Is Recognized as an Independent Condition by an International Classification of Disease, Tenth Revision, Clinical Modification (ICD-10-CM) Code. J Am Med Dir Assoc.

[4] Dennison EM, Sayer AA, Cooper C (2017). Epidemiology of sarcopenia and insight into possible therapeutic targets. Nat Rev Rheumatol.

[5] Cruz-Jentoft AJ (2010). Sarcopenia: European consensus on definition and diagnosis: Report of the European Working Group on Sarcopenia in Older People. Age and ageing.

[6] Guglielmi G (2016). The role of DXA in sarcopenia. Aging Clin Exp Res.

[7] Janssen I, Heymsfield SB, Baumgartner RN, Ross R (2000). Estimation of skeletal muscle mass by bioelectrical impedance analysis. J Appl Physiol (1985).

[8] Scharf G, Heineke J (2012). Finding good biomarkers for sarcopenia. J Cachexia Sarcopenia Muscle.

[9] Kalinkovich A, Livshits G (2015). Sarcopenia–The search for emerging biomarkers. Ageing Res Rev.

[10] Cruz-Jentoft AJ (2014). Prevalence of and interventions for sarcopenia in ageing adults: a systematic review. Report of the International Sarcopenia Initiative (EWGSOP and IWGS). Age and ageing.

[11] Ilich JZ (2014). Interrelationship among muscle, fat, and bone: connecting the dots on cellular, hormonal, and whole body levels. Ageing Res Rev.

[12] Kob R (2015). Sarcopenic obesity: molecular clues to a better understanding of its pathogenesis?. Biogerontology.

[13] Marzetti E (2014). Serum levels of C-terminal agrin fragment (CAF) are associated with sarcopenia in older hip fractured patients. Exp Gerontol.

[14] Sakuma K, Aoi W, Yamaguchi A (2015). Current understanding of sarcopenia: possible candidates modulating muscle mass. Pflugers Arch.

[15] Lauretani F (2003). Age-associated changes in skeletal muscles and their effect on mobility: an operational diagnosis of sarcopenia. J Appl Physiol (1985).

[16] Chen LK (2014). Sarcopenia in Asia: consensus report of the Asian Working Group for Sarcopenia. J Am Med Dir Assoc.

[17] Kim SK (2014). A nineteen gene-based risk score classifier predicts prognosis of colorectal cancer patients. Mol Oncol.

[18] Kim SM (2012). Sixty-five gene-based risk score classifier predicts overall survival in hepatocellular carcinoma. Hepatology.

[19] Sumukadas D, Struthers AD, McMurdo ME (2006). Sarcopenia–a potential target for Angiotensin-converting enzyme inhibition?. Gerontology.

[20] Perrini S (2010). The GH/IGF1 axis and signaling pathways in the muscle and bone: mechanisms underlying age-related skeletal muscle wasting and osteoporosis. J Endocrinol.

[21] Berry SD (2013). Procollagen type III N-terminal peptide (P3NP) and lean mass: a cross-sectional study. J Frailty Aging.

[22] Izumiya Y (2008). FGF21 is an Akt-regulated myokine. FEBS Lett.

[23] Lee SJ (2004). Regulation of muscle mass by myostatin. Annu Rev Cell Dev Biol.

[24] Sinha M (2014). Restoring systemic GDF11 levels reverses age-related dysfunction in mouse skeletal muscle. Science.

[25] Rao RR (2014). Meteorin-like is a hormone that regulates immune-adipose interactions to increase beige fat thermogenesis. Cell.

[26] Reimann J (2010). Macrophage migration inhibitory factor in normal human skeletal muscle and inflammatory myopathies. J Neuropathol Exp Neurol.

[27] Pritt ML (2008). Fabp3 as a biomarker of skeletal muscle toxicity in the rat: comparison with conventional biomarkers. Toxicol Sci.

[28] Guillet C, Auguste P, Mayo W, Kreher P, Gascan H (1999). Ciliary neurotrophic factor is a regulator of muscular strength in aging. J Neurosci.

[29] Nakamura, K., Yamanouchi, K. & Nishihara, M. [Transdisciplinary Approach for Sarcopenia. Molecular mechanism of sarcopenia: The role of skeletal muscle niche component SPARC in the regulation of myogenesis and adipogenesis and its alteration with age]. *Clin Calcium* **24**, 1471–1478, CliCa141014711478 (2014). 25266092

[30] Peake JM, Della Gatta P, Suzuki K, Nieman DC (2015). Cytokine expression and secretion by skeletal muscle cells: regulatory mechanisms and exercise effects. Exerc Immunol Rev.

[31] Pedersen BK, Febbraio MA (2008). Muscle as an endocrine organ: focus on muscle-derived interleukin-6. Physiol Rev.

[32] Lane TF, Sage EH (1994). The biology of SPARC, a protein that modulates cell-matrix interactions. FASEB J.

[33] Yan Q, Sage EH (1999). SPARC, a matricellular glycoprotein with important biological functions. J Histochem Cytochem.

[34] Termine JD (1981). Osteonectin, a bone-specific protein linking mineral to collagen. Cell.

[35] Jorgensen LH (2009). Secreted protein acidic and rich in cysteine (SPARC) in human skeletal muscle. J Histochem Cytochem.

[36] Petersson SJ (2013). SPARC is up-regulated during skeletal muscle regeneration and inhibits myoblast differentiation. Histol Histopathol.

[37] Nakamura K, Nakano S, Miyoshi T, Yamanouchi K, Nishihara M (2013). Loss of SPARC in mouse skeletal muscle causes myofiber atrophy. Muscle Nerve.

[38] Serre-Beinier V (2010). Macrophage migration inhibitory factor deficiency leads to age-dependent impairment of glucose homeostasis in mice. J Endocrinol.

[39] Goldspink G (2007). Loss of muscle strength during aging studied at the gene level. Rejuvenation Res.

[40] Stitt TN (2004). The IGF-1/PI3K/Akt pathway prevents expression of muscle atrophy-induced ubiquitin ligases by inhibiting FOXO transcription factors. Mol Cell.

[41] Jennische E, Skottner A, Hansson HA (1987). Satellite cells express the trophic factor IGF-I in regenerating skeletal muscle. Acta Physiol Scand.

[42] Pelosi L (2007). Local expression of IGF-1 accelerates muscle regeneration by rapidly modulating inflammatory cytokines and chemokines. FASEB J.

[43] Jacquemin V, Furling D, Bigot A, Butler-Browne GS, Mouly V (2004). IGF-1 induces human myotube hypertrophy by increasing cell recruitment. Exp Cell Res.

[44] Chung HY (2009). Molecular inflammation: underpinnings of aging and age-related diseases. Ageing Res Rev.

