# Supplementary material for: Prediction of sarcopenia using a combination of multiple serum biomarkers
Source: Sci Rep. 2018 Jun 5;8:8574. doi: 10.1038/s41598-018-26617-9 (PMC5988732; doi:10.1038/s41598-018-26617-9)
Supplement: Supplementary file 1 — Supplementary Figure 1. [file 41598_2018_26617_MOESM1_ESM.pdf]

Prediction of sarcopenia using a combination of multiple serum biomarkers

Ju Yeon Kwak[1†], Hyeoncheol Hwang[2†], Seon-Kyu Kim[3†], Jeong Yi Choi[1], Seung-Min Lee[1], Hyun Bang[2], Eun-Soo Kwon[1], Kwang-Pyo Lee[1, 5], Sun Gun Chung[2, 4*], and Ki-Sun Kwon[1, 6*]

[1]*Aging Research Center, Korea Research Institute of Bioscience & Biotechnology, 125 Gwahak-ro, Yuseong-gu, Daejeon, 34141, Republic of Korea.* [2]*Department of Rehabilitation Medicine, Seoul National University College of Medicine, Seoul National University Hospital, 101 Daehak-ro, Jongno-gu, Seoul, 03080, Republic of Korea.* [3]*Personalized Genomic Medicine Research Center, Korea Research Institute of Bioscience & Biotechnology, 125 Gwahak-ro, Yuseong-gu, Daejeon, 34141, Republic of Korea.* [4]*Institute of Aging, Seoul National University, 101 Daehak-ro, Jongno-gu, Seoul, 03080, Republic of Korea.* [5]*Department of Biomolecular Science, and* [6]*Functional Genomics, Korea University of Science and Technology (UST), 217 Gajeong-ro, Yuseong-gu, Daejeon, 34113, Republic of Korea*

[†]equal contribution

[*]Corresopond to:

Ki-Sun Kwon, Korea Research Institute of Bioscience and Biotechnology, 125 Gwahak-ro, Yuseong-gu, Daejeon, 34141, Republic of Korea, Tel.: +82-42-860-4143, Fax: +82-42-879-8596, E-mail: kwonks@kribb.re.kr

Sun Gun Chung, Department of Rehabilitation Medicine, College of Medicine, Seoul National University, 101 Daehak-ro, Jongno-gu, Seoul, 03080, Republic of Korea, Tel: +82-2-2072-2560, Fax: +82-2-743-7473, E-mail: suncg@snu.ac.kr

Supplementary Figure 1.

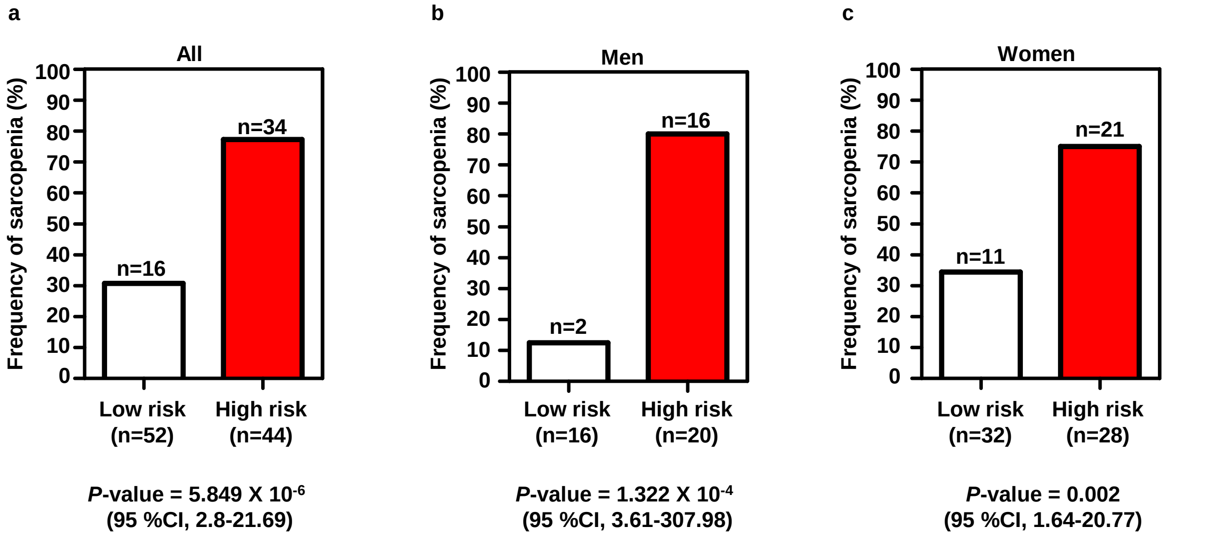

Supplementary Figure 1. Comparison of frequencies of sarcopenia based on ROC analysis.

(a) Comparison of frequencies of sarcopenia in low- and high-risk groups stratified by best cutoff (1.529) obtained from ROC analysis. Bar plots displaying sarcopenia frequencies of low- and high-risk groups in men (b) and women (c). The best cutoff values in men and women were calculated as 1.543 and 1.505, respectively. *P*-value and confidence interval (CI) were obtained using Fisher's exact test.
